# Absence of evidence is not evidence of absence

**DOI:** 10.1590/1678-7757-2023-ed001

**Published:** 2023-03-27

**Authors:** Magda FERES, Murilo Fernando Neuppmann FERES

**Affiliations:** 1 Harvard School of Dental Medicine, Infection and Immunity Department of Oral Medicine Boston Massachusetts USA Harvard School of Dental Medicine, Infection and Immunity, Department of Oral Medicine, Boston, Massachusetts, USA.; 2 Universidade Guarulhos Divisão de Pesquisa em Odontologia Departamento de Periodontologia Guarulhos São Paulo Brasil Universidade Guarulhos, Divisão de Pesquisa em Odontologia, Departamento de Periodontologia, Guarulhos, São Paulo, Brasil.; 3 The Forsyth Institute Cambridge Massachusetts USA The Forsyth Institute, Cambridge, Massachusetts, USA.; 4 Universidade de São Paulo Faculdade de Odontologia de Ribeirão Preto Departamento de Odontopediatria Ribeirão Preto São Paulo Brasil Universidade de São Paulo, Faculdade de Odontologia de Ribeirão Preto, Departamento de Odontopediatria, Ribeirão Preto, São Paulo, Brasil.

“Absence of Evidence is not Evidence of Absence” is a quote by Carl Sagan, an American astronomer and one of the leading science communicators of the 20^th^ century.^1^ Its importance relies on the highlight of the logical fallacy where a hypothesis is assumed to be true or false before being scientifically and satisfactorily investigated. With that in mind, in this editorial, we comment on how this premise directly applies to clinical practice.

When a certain treatment modality has already been investigated and proven superior to another one or even to traditional therapy, the clinical decision is straightforward. As a result, we should select this modality over others and apply it to our patients, as its benefits are unambiguously supported by reliable scientific proof.^2^ However, several treatments lack sufficient evidence to justify an upfront recommendation without further consideration. In this case, we must understand what kind of “absence of evidence” we are dealing with. Two scenarios may apply.

The first one is when we are dealing with a therapy that has been extensively tested but failed to show beneficial results when compared with others. In this case, the recommendation should be not to use it. Let’s consider a practical example related to periodontitis. Several studies have evaluated the effects of systemic doxycycline as an adjunct in the treatment of patients with severe periodontitis. Nevertheless, these studies have failed to show a consistent benefit of this agent,^3^ whereas other antibiotic protocols have demonstrated to be more effective than doxycycline.^4 - 6^ Therefore, this treatment should not be selected over others, as it has already been tested and showed no benefits to justify its use.

The second scenario – the one addressed in the quotation in question – is when we lack reliable evidence for recommending or not a specific therapy because it has not yet been comprehensively evaluated by clinical trials. In this case, we cannot simply assume that the treatment is effective or not. In other words, lacking evidence does not imply any benefit conclusions of a specific therapy or even the existence of a benefit itself. Here, the decision should also be not to recommend the treatment.

Neither few, nor poorly designed studies, nor investigations enrolling small samples, nor research resulting in clinically irrelevant differences should ever support clinical decision-making. Yet, in some circumstances, such as clinical conditions that may incur risk for the patient’s general health (e.g., post-surgical infection) or harmless therapies supported by moderate evidence (e.g., natural products), the clinical decision may be guided by three main factors: (1) “Biological Plausibility,” (2) Risk/Benefit Analysis, and (3) Cost/Benefit Analysis.

For (1) “biological plausibility,” examples of clinical situations include using or not an antibiotic after conducting a connective gingival graft or a periodontal regeneration procedure, or after the placing of a single implant.^7^ Here, the “biological plausibility” for prescribing the antibiotics should consider (i) the type and duration of the surgical procedure, (ii) if the surgery involved bone removal and extension of the osteotomy, and others.

Regarding (2) the risk/benefit analysis, one should consider (i) the direct risk for the patient, (ii) the risk for the population, and (iii) the risk for the environment. For example, when dealing with antibiotics, we must consider the risk to the patient (i.e., side effects), as well as to society as a whole. The indiscriminate use of antibiotics may increase bacterial resistance to these drugs in the general population and decrease their effectiveness, including for lethal diseases.^8^ Finally, one should also evaluate (3) the cost/benefit balance before choosing a certain treatment, that is, what is the investment for both patient and practitioner to apply a therapy that has not been proven to have a real clinical benefit – even for unhazardous treatments. The cost-benefit analysis should be applied to any clinical question, especially when dealing with high-cost therapies, such as those involving some biomaterials or expensive laser equipment.^9^

The aforementioned reasoning entails how to approach every situation when deciding which is the best treatment protocol for our patients. Our concern about the dental field is that we often see treatments that are not evidence-based being widely used in clinical practice^10 - 11^– some of them even being recommended as “established protocols.” On the other hand, protocols that have been already supported by reliable scientific evidence may take too long to be incorporated into clinical practice.^12 - 13^

Researchers and clinicians must understand which therapies are proven effective or ineffective, and differentiate them from those that still need to be properly investigated. This will help support effective clinical practice and identify gaps in knowledge. To achieve this goal, we must advance our understanding of clinical trials and systematic/scoping reviews of clinical and laboratory data.^14^ Finding, discriminating, and interpreting scientific literature is one of the most relevant abilities of a health care professional,^15^ and we should pursue this goal!
